# TGF-β Signaling Regulates SLC8A3 Expression and Prevents Oxidative Stress in Developing Midbrain Dopaminergic and Dorsal Raphe Serotonergic Neurons

**DOI:** 10.3390/ijms21082735

**Published:** 2020-04-15

**Authors:** Enaam Chleilat, Abhishek Pethe, Dietmar Pfeifer, Kerstin Krieglstein, Eleni Roussa

**Affiliations:** 1 Institute of Anatomy and Cell Biology, Department of Molecular Embryology, Faculty of Medicine, University of Freiburg, Albertstrasse 17, 79104 Freiburg, Germany; enaam.chleilat@universitaets-herzzentrum.de (E.C.); abhishek.pethe@anat.uni-freiburg.de (A.P.); Kerstin.krieglstein@anat.uni-freiburg.de (K.K.); 2 Department of Hematology, Oncology and Stem Cell Transplantation, University Medical Center Freiburg, University of Freiburg, 79106 Freiburg, Germany; dietmar.pfeifer@uniklinik-freiburg.de

**Keywords:** calcium homeostasis, ROS, NCX3, growth factor, neuronal development, hindbrain

## Abstract

Calcium homeostasis is a cellular process required for proper cell function and survival, maintained by the coordinated action of several transporters, among them members of the Na^+^/Ca^2+^-exchanger family, such as SLC8A3. Transforming growth factor beta (TGF-β) signaling defines neuronal development and survival and may regulate the expression of channels and transporters. We investigated the regulation of SLC8A3 by TGF-β in a conditional knockout mouse with deletion of TGF-β signaling from Engrailed 1-expressing cells, i.e., in cells from the midbrain and rhombomere 1, and elucidated the underlying molecular mechanisms. The results show that SLC8A3 is significantly downregulated in developing dopaminergic and dorsal raphe serotonergic neurons in mutants and that low SLC8A3 abundance prevents the expression of the anti-apoptotic protein Bcl-xL. TGF-β signaling affects SLC8A3 via the canonical and p38 signaling pathway and may increase the binding of Smad4 to the *Slc8a3* promoter. Expression of the lipid peroxidation marker malondialdehyde (MDA) was increased following knockdown of *Slc8a3* expression in vitro. In neurons lacking TGF-β signaling, the number of MDA- and 4-hydroxynonenal (4-HNE)-positive cells was significantly increased, accompanied with increased cellular 4-HNE abundance. These results suggest that TGF-β contributes to the regulation of SLC8A3 expression in developing dopaminergic and dorsal raphe serotonergic neurons, thereby preventing oxidative stress.

## 1. Introduction

Members of the Transforming growth factor beta (TGF-β) family are established molecular players in regulating several cellular processes during development, in health and disease [1,2,3]. During neuronal development, TGF-βs are indispensable molecular determinants for the differentiation of several neuronal populations, among them dopaminergic and serotonergic neurons. Recent studies using animal models with cell type-specific deletion of either TGF-β ligands or TGF-β signaling have shown severe impairment in the differentiation of individual midbrain dopaminergic and hindbrain serotonergic subpopulations, a process associated with increased neuronal cell death [4,5].

Cellular ionic homeostasis is a prerequisite for proper cellular function and survival, whereby TGF-βs are known to regulate several channels and transporters within or outside the central nervous system [6,7,8,9]. Particularly, the link between TGF-βs and Ca^2+^ homeostasis has been documented in several cellular paradigms: In cortical neurons, TGF-β regulates L-type Ca^2+^ channels through MEK, JNK1/2, and p38 MAPK signaling [10]; it increases store-operated Ca^2+^ entry into megakaryocytes [11]; and it enhances Ca^2+^ influx pathways and the expression of transient receptor potential canonical channels (TRPCs) in human cardiac fibroblasts. Interestingly, human cardiac fibroblasts express several TRPC-mediated Ca^2+^ influx pathways, which activate the reverse mode Na^+^/Ca^2+^ exchanger (NCX) [12].

Among the NCX isoformsSLC8A3), the isoform 3 of the Na^+^/Ca^2+^ exchanger (NCX3), is exclusively expressed in excitable cells [13]. It mediates the electrogenic transport of Na^+^ and Ca^2+^, and contributes to the maintenance of Ca^2+^ homeostasis. SLC8A3 acts with a stoichiometry of 3:1 and may operate in the forward (Ca^2+^ efflux) or reverse (influx of Ca^2+^) mode. However, the forward mode is the predominant mode of Na^+^/Ca^2+^ exchanger action. Alternative splicing generates two variants, AC and B, which display a tissue-specific distribution in mice. The variant B of *Slc8a3* is mostly expressed in the brain, including substantia nigra pars compacta (SNc) and hindbrain raphe nuclei, whereas the variant AC is predominant in skeletal muscle. The functional significance of SLC8A3 has been appreciated in many studies, as reviewed by Michel et al. (2015) [14]. The capacity of handling Ca^2+^ during excitotoxicity in neurons has been exclusively attributed to SLC8A3, whereas during brain development, SLC8A3 contributes to the maturation of oligodendrocytes [15]. Mice deficient for *Slc8a3* are viable, but they show skeletal muscle fiber necrosis and impaired neuromuscular transmission, associated with reduced motor activity, weakness of the forelimb muscles, and fatigability [16]. Moreover, *Slc8a3*-deficient mice show impaired hippocampal long-term potentiation and spatial learning [17]. Cortical neurons and hippocampal organotypic cultures from *Slc8a3*-deficient mice display considerable neuronal death when exposed to oxygen glucose deprivation and reoxygenation [17,18,19].

Notably, SLC8A3 can be regulated by neurotrophic factors. Indeed, in differentiating neurons, *Slc8a3* promoter activity can be enhanced, beyond Ca^2+^ and retinoid acid, but also by brain-derived neurotrophic factor (BDNF) [20]. It has been proposed that SLC8A3 might play a crucial role in neuronal differentiation and neuronal function. Furthermore, in PC12 cells, nerve growth factor (NGF) increases both isoform 1 and isoform 3 of the Na^+^/Ca^2+^ exchanger (Formisano et al., 2008) [21]. It has also been shown that SLC8A3 basal expression, as well as NGF-induced upregulation of SLC8A3 are regulated by MEK1 (Sirabella et al., 2012) [22].

In the present study, we made use of a mouse line with conditional deletion of TGF-β signaling from Engrailed 1 (En1)-expressing cells to investigate the regulation of SLC8A3 in differentiating midbrain dopaminergic and dorsal raphe hindbrain serotonergic neurons. The results show significant downregulation of SLC8A3 in mutants, compared to wild type. We also show a putative regulation of Smad4 binding to *Slc8a3* promoter via TGF-β and that low SLC8A3 abundance prevents the expression of the anti-apoptotic Bcl-xL [23]. In neurons lacking TGF-β signaling, the number of malondialdehyde (MDA)- and 4-hydroxynonenal (4-HNE) positive cells was significantly increased, accompanied with an increased cellular 4-HNE abundance.

## 2. Results

### 2.1. SLC8A3 Expression is Regulated by TGF-β Signaling

In a previous study, we have shown a phenotype in the midbrain and ventral hindbrain of *TβRII^flox/flox^::En1^cre/+^* animals at embryonic day (E) 16–18. The number of midbrain dopaminergic neurons and dorsal raphe serotonergic neurons was significantly decreased in conditional knock out (*cKO)* animals, compared to wild type (*wt)*, a phenotype accompanied by significantly increased neuronal cell death [4]. 

Impaired Ca^2+^ homeostasis may underlie neuronal cell death and possibly may be responsible for the neuronal loss observed at E16 in *TβRII^flox/flox^::En1^cre/+^*. Among the genes that mediate calcium homeostasis, *Slc8a3*, the isoform 3 of the Na^+^/Ca^2+^ exchanger, is predominantly expressed in substantia nigra dopaminergic neurons and mitochondrial SLC8A3 prevents neuronal degeneration induced by mitochondrial Ca^2+^ accumulation through interaction with PTEN-induced kinase 1 (PINK) [24].

**Figure 1:**
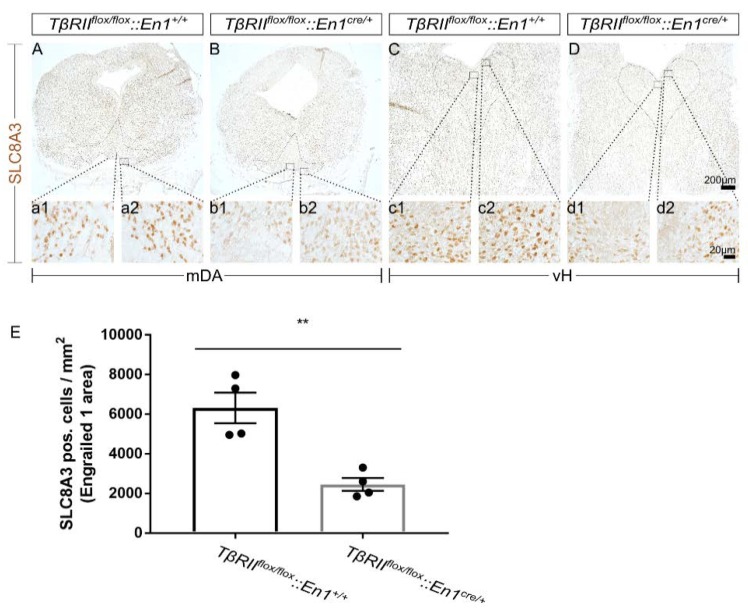
Impaired SLC8A3 expression by loss of TGF-β signaling. (**A**–**D**): Immunoperoxidase light microscopy for SLC8A3 on fixed coronal sections from the mouse midbrain (**A**,**B**) and ventral hindbrain (vH; **C**,**D**) of wild type (*wt)* (**A**,**C**) and conditional knock out (*cKO)* (**B**,**D**) at embryonic day 16 shows a decreased labelling intensity in the area of midbrain dopaminergic neurons (a1, a2, b1, and b2 are a higher magnification of the black-boxed areas in **A** and **B**) and hindbrain dopaminergic neurons (c1, c2, d1, and d2 are a higher magnification of the black-boxed areas in **C** and **D**) in *cKO*, compared to *wt*. (**E**) Quantification of SLC8A3-positive cells in the Engrailed 1 area/mm^2^. ** *p* < 0.01, using the two-tailed unpaired Student’s *t*-test, *n* = 4/genotype).

To test whether impaired SLC8A3 expression might contribute to the phenotype observed in *TβRII^flox/flox^::En1^cre/+^,* first, we determined the SLC8A3 protein expression in the midbrain dopaminergic (mDA) and ventral hindbrain (vH) serotonergic area in *TβRII^flox/flox^::En1^cre/+^*, by immunohistochemistry at E16 using an antibody against SLC8A3. As shown in Figure 1A–D, in *wt*, SLC8A3 was broadly distributed in both the ventral midbrain (Figure 1A) and hindbrain (Figure 1C), consistent with previous observations [24]. Immunolabeling of a moderate to strong intensity was detected in neurons of the substantia nigra pars compacta (SNc; a2), ventral tegmental area (VTA; a1), and all subpopulations of the dorsal raphe (DR; c1 and c2). In *TβRII^flox/flox^::En1^cre/+^* animals, however, both the cell number of immunopositive neurons and labeling intensity were considerably decreased in both mDA (B, b1, and b2 for VTA and SNc, respectively) and serotonergic (5-HT) neurons of the dorsal raphe (D, d1, and d2). Indeed, quantification of SLC8A3-positive neurons showed a significant decreased number within the Engrailed 1 area, encompassing both the dorsomedial DR (B7) and the caudal VTA (A10), in *cKO* embryos, compared to *wt* littermates (Figure 1E; 6312 ± 775.6 and 2452 ± 325.9, for *wt* and *cKO*, respectively, ** *p* < 0.01, using the two-tailed unpaired Student’s *t*-test, *n* = 4)

### 2.2. Anti-Apoptotic Action of SLC8A3

**Figure 2:**
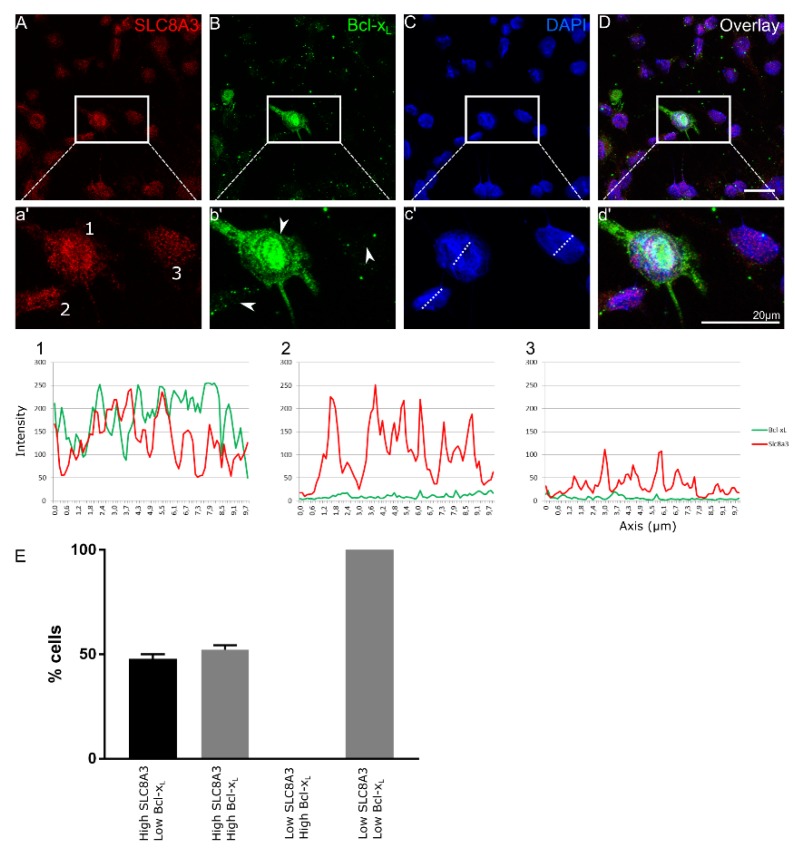
SLC8A3 and Bcl-xL expression correlate in developing Engrailed 1-derived midbrain and hindbrain neurons. (**A**–**D**) Confocal double immunofluorescent representative labelling of SLC8A3 (red) and the anti-apoptotic marker Bcl-xL (green) in primary *TβRII^flox/flox^::En1^cre/+^* neuronal ventral midbrain/hindbrain cultures at E14 show either high or low SLC8A3 expression and high or low Bcl-xL expression. Line scan (1) is a representative image of a neuron with high SLC8A3 expression and high Bcl-xL expression (neuron 1 in the magnification of the white boxed area). Line scan (2) depicts neurons with high SLC8A3 expression while Bcl-xL is low (neuron 2 in the magnification of the white boxed area). Line scan (3) shows low cellular SLC8A3 abundance together with low Bcl-xL (neuron 3 in the magnification of the white boxed area). Arrowheads correspond to cells 1-3. (**E**) Quantification of number of cells (%) based on high/low SLC8A3 and high/low Bcl-xL expression (total: 96 cells from 3 independent experiments).

We have previously shown increased neuronal cell death accompanied by increased caspase 3-positive cells in *TβRII^flox/flox^::En1^cre/+^* animals at E16 [4]. We hypothesized that impaired SLC8A3 expression contributes to the observed increased neuronal cell death in the *cKO*. Therefore, we performed double immunofluorescence in primary neuronal cultures from *cKO* at E14 for SLC8A3 and the anti-apoptotic marker Bcl-xL [23,25] (Figure 2A–D). We scored the cells as those with either “high” or “low” SLC8A3 abundance (Representative cells 1-3 in a’, arrowheads in b’). As shown in the magnification of the white boxed areas (2a’–2d’) and representative line scans, high SLC8A3 expression was associated either with high Bcl-xL abundance (52.2 ± 2.2%; line scan 1) or with low Bcl-xL expression (47.8 ± 2.2%; line scan 2), whereas in low SLC8A3-expressing cells (line scan 3), expression of Bcl-xL was either low or absent. As shown in Figure 2E, there is a statistically significant difference on the Bcl-xL intensity across ventral midbrain/hindbrain neurons between high and low SLC8A3-expressing cell groups. These results imply that low SLC8A3 abundance prevents the expression of the anti-apoptotic protein Bcl-xL and may therefore represent a crucial molecular component for neuronal survival.

### 2.3. Cell-Type-Dependent Regulation of SLC8A3 by TGF-β 

**Figure 3:**
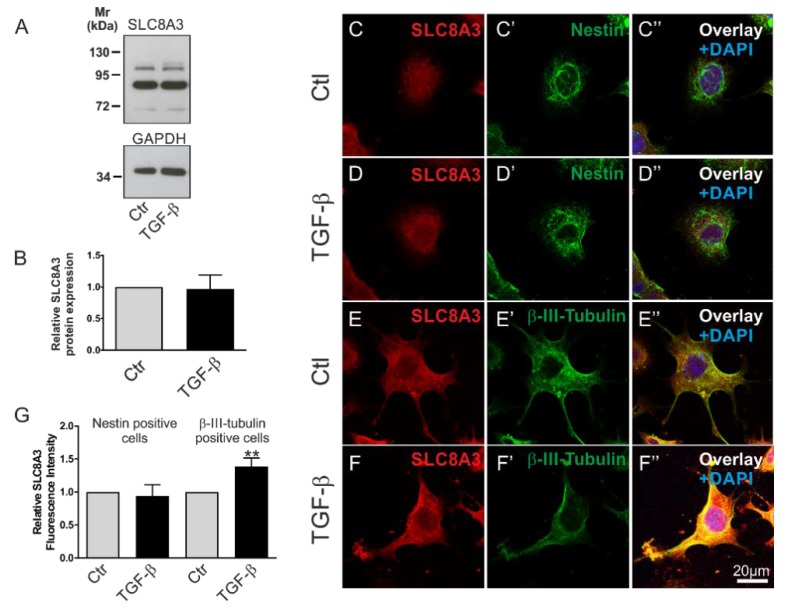
Differential regulation of SLC8A3 in Nestin-positive cells and neurons by TGF-β. (**A**) Protein abundance of SLC8A3 by immunoblotting in control MN9D cells in the presence of exogenous recombinant TGF-β (2 ng/mL), 30 µg protein was loaded per lane. (**B**) Quantification, not significant after densitometric analysis of the signal ratio SLC8A3:GAPDH and two-tailed unpaired Student’s *t*-test, *n* = 3. The value of control (ctl) was set to 1. (**C**–**F″**): Confocal double immunofluorescence for SLC8A3 (red) with the stem cell marker Nestin (green; **C’**,**D’**) or the neuronal marker β-III tubulin (green; **E’**,**F’**) in MN9D cells in the presence (**D**,**F**) or absence (**C**,**E**) of TGF-β. (**G**) Quantification of SLC8A3 immunofluorescence intensity in Nestin-positive cells and β-III-tubulin-positive cells in controls and following TGF-β treatment. ** *p* < 0.01, using two-tailed unpaired Student’s *t*-test, 30–100 cells/experimental group from 3 to 7 independent experiments. The value of control (ctl) was set to 1.

To investigate whether the activation of TGF-β signaling regulates SLC8A3, we first tested the expression of type II TGF-β receptor (TβRII) in the Lund human mesencephalic (LUHMES) and the mouse MN9D cell lines [26,27]. As shown in the Supplementary Materials, using an antibody against TβRII, multiple bands were detected in whole cell homogenates from both MN9D and LUHMES cells. Depending on the species, splice variant, and glycosylation state, the expected size of TβRII ranges between 63 and 85 kDa. Among other immunoreactive bands, prominent bands at ~65 and ~70 kDa (arrows) were detected in MN9D and LUHMES cells, respectively. We further asked whether TGF-β signaling might affect the protein abundance of SLC8A3. Therefore, MN9D cells were treated with exogenous TGF-β (2 ng/mL) for 60 min, and subsequently SLC8A3 protein abundance was determined by immunoblot analysis (Figure 3A). Using an antibody raised against SLC8A3, an immunoreactive band at ~103 kDa, a weaker one at ~110 kDa, and a prominent band at ~85 kDa were detected in the controls, and treatment of the cells with TGF-β for 60 min had no significant effect on the SLC8A3 abundance (Figure 3B, 0.96 ± 0.22 fold, not significant, *n* = 3 using the two tailed unpaired Student’s *t*-test. The ~103110 kDa bands, which correspond to the molecular mass of SLC8A3, were quantified). The same results were also obtained using the LUHMES cell line (data not shown). However, MN9D cells are heterogeneous, comprising of progenitor cells and differentiated neurons. To investigate SLC8A3 protein expression in these two cell populations separately, we performed double immunofluorescence for SLC8A3 with the stem cell marker Nestin and the neuronal marker β-III-tubulin. As shown in Figure 3C–D″,G, the abundance of SLC8A3 protein in Nestin-positive cells was low (Figure 3C) and treatment with exogenous TGF-β (Figure 3D) had no effect on SLC8A3 immunofluorescence intensity (Figure 3G; 0.94 ± 0.17 fold, compared to the untreated controls, not significant, using two-tailed unpaired Student’s *t*-test; *n* = 3). In contrast, differentiated neurons exhibited obvious SLC8A3 immunolabeling (Figure 3E) and treatment with exogenous TGF-β (Figure 3F) significantly increased SLC8A3 labeling intensity (Figure 3G; 1.39 ± 0.13 fold, compared to the untreated controls, ** *p* < 0.01, using two-tailed unpaired Student’s *t*-test, *n* = 7). These data suggest that TGF-β differentially regulates SLC8A3 abundance in cells at distinct developmental stages.

### 2.4. TGF-β Canonical Pathway and p38 Signaling Pathway Regulate Basal SLC8A3 Protein Expression

**Figure 4:**
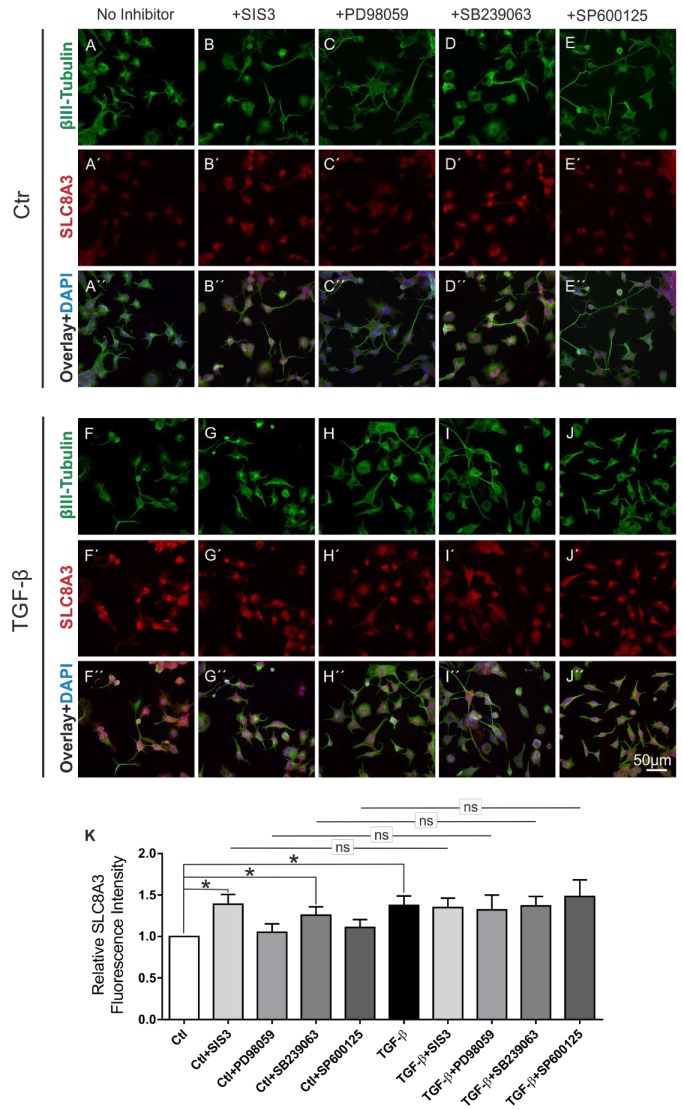
TGF-β canonical pathway and p38 pathway regulate basal SLC8A3 levels. Confocal double immunofluorescence for SLC8A3 (red) with the neuronal marker β-III tubulin (green) in control (ctl; untreated) (**A**–**E**) and TGF-β-treated (**F**–**J**) MN9D cells for 60 min following application of the inhibitor of Smad3 SIS3 (3 µM; **B**–**B‴**,**G**–**G‴**), or the MAP kinase inhibitor PD98059 (25 µM; **C**–**C‴**,**H**–**H‴**), or the p38 inhibitor SB239063 (10 µM; **D**–**D‴**,**I**–**I‴**), or the inhibitor of JNK signaling SP600125 (10 µM; **E**–**E‴**,**J**–**J‴**), (**K**) Quantification of SLC8A3 fluorescence intensity, * *p* < 0.05, ** *p* < 0.01, using the two-tailed unpaired Student’s t-test or one-way ANOVA and Bonferroni post hoc test, total 979 cells analyzed from 5 independent experiments. The values of control were set to 1.

MAPKs regulate NCX expression in an NCX-isoform and in a MAPK cascade-specific manner. In PC12 cells, upon NGF stimulation, both ERK1/2 and p38 upregulated NCX3 [22]. To elucidate the pathway by which TGF-β signaling regulates SLC8A3, control and TGF-β-treated (2 ng/mL) MN9D cells were incubated for 60 min in the presence or absence of the following inhibitors: Specific Smad3 inhibitor SIS3 (3 µM) for blocking the canonical pathway, JNK inhibitor SP600125 (10 µM), MEK1 and MEK2 inhibitor PD98059 (25 µM), or the p38 inhibitor SB239063 (10 µM). Subsequently, double immunofluorescence for β-III-tubulin and SLC8A3 was performed (Figure 4). In control untreated cells (ctl), blocking of either the MEK1/2 or JNK pathway had no effect on SLC8A3 immunolabeling, as shown in Figure 4A–A‴,C–C‴,D–D‴,K (1.05 ± 0.1 fold and 1.10 ± 0.09 fold, for inhibition with PD98059 and SP600125, respectively; not significant). Surprisingly, blocking of the canonical, i.e., Smad dependent, pathway by SIS3 (Figure 4B–B‴) or inhibiting the p38 signaling (Figure 4E–E‴) significantly increased SLC8A3 fluorescence intensity, compared to untreated cells (Figure 4K: 1.39 ± 0.11 fold and 1.25 ± 0.10 fold for SIS3 and SB239063, respectively; * *p* < 0.05, using the two-tailed unpaired Student’s *t*-test, 84–126 cells/experimental group from *n* = 5). In TGF-β-treated MN9D cells (Figure 4F–F‴), SLC8A3 was upregulated in β-III-tubulin-positive cells (1.37 ± 0.11 fold; * *p* < 0.05) and inhibition of any pathway had no effect on the TGF-β-dependent increased SLC8A3 abundance (Figure 4F–J‴,K, 1.35 ± 0.11 fold, 1.32 ± 0.18 fold, 1.37 ± 0.11 fold, 1.48 ± 0.20 fold, for SIS3, PD98059, SB239063, and SP6000125, respectively * *p* < 0.05, compared to the untreated controls, and not significant compared to the TGF-β treatment alone, using two-tailed unpaired Student’s *t*-test and one-way ANOVA and Bonferroni post-hoc test, 83–107 cells/experimental group, *n* = 5).

These data suggest that the TGF-β canonical and p38 signaling pathways likely regulate basal SLC8A3 protein levels.

### 2.5. Smad4 Binds to Slc8a3 Promoter

**Figure 5:**
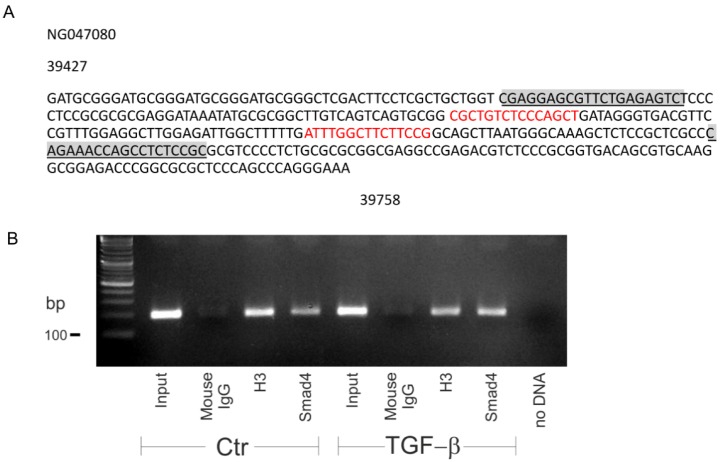
Smad4 specifically binds to the promoter of *Slc8a3*. (**A**) Smad4 binding site(s) (red) to the *Slc8a3* promoter region. Underlined sequences represent primer sequences used to detect Smad4 binding. (**B**) PCR following the chromatin immunoprecipitation assay of the control and TGF-β-treated (2 ng/mL for 30 min) Lund human mesencephalic (LUHMES) cells demonstrates increased binding of Smad4 to the predicted binding sequence in the promoter region of *Slc8a3*. Predicted PCR product size 193 bp. Histone 3 (H3) and mouse IgG represent the positive and negative control, respectively. The figure is a representative of *n* = 2.

Having shown that the canonical pathway regulates basal SLC8A3, we next asked whether TGF-β directly regulates *Slc8a3.* Therefore, we performed a search for conserved Smad binding sequences in the promoter region of *Slc8a3* (Figure 5A). Indeed, the *Slc8a3* promoter on chromosome 14 contains two conserved Smad4 binding sites (sequences in red). To demonstrate that Smad4 binds to the *Slc8a3* promoter sequence, chromatin immunoprecipitation (ChIP) was performed in control LUHMES cells and in LUHMES cells after treatment with exogenous TGF-β (2 ng/mL) for 30 min. Smad4 was immunoprecipitated using an antibody against Smad4, and subsequently PCR was performed to amplify DNA fragments bound to Smad4. Therefore, primers flanking the Smad4 binding sequences were used, as shown in Figure 5A (underlined sequences). The results are shown in Figure 5B. Bands at the expected size of 193 bp were detected in the input of the control (lane 1) and treated LUHMES cells (lane 5). Moreover, a band was also detected for the Smad4 binding sequence in the *Slc8a3* promoter region following TGF-β treatment (lane 8), which was more prominent, compared to that in control LUHMES cells (lane 4). Histone 3 (H3) (lanes 3 and 7) and IgG (lanes 2 and 6) were used as a positive and negative control for the immunoprecipitation, respectively. These results imply that Smad4 binding to the *Slc8a3* promoter may be regulated by TGF-β.

### 2.6. Knockdown of Slc8a3 is Associated with Oxidative Stress

**Figure 6:**
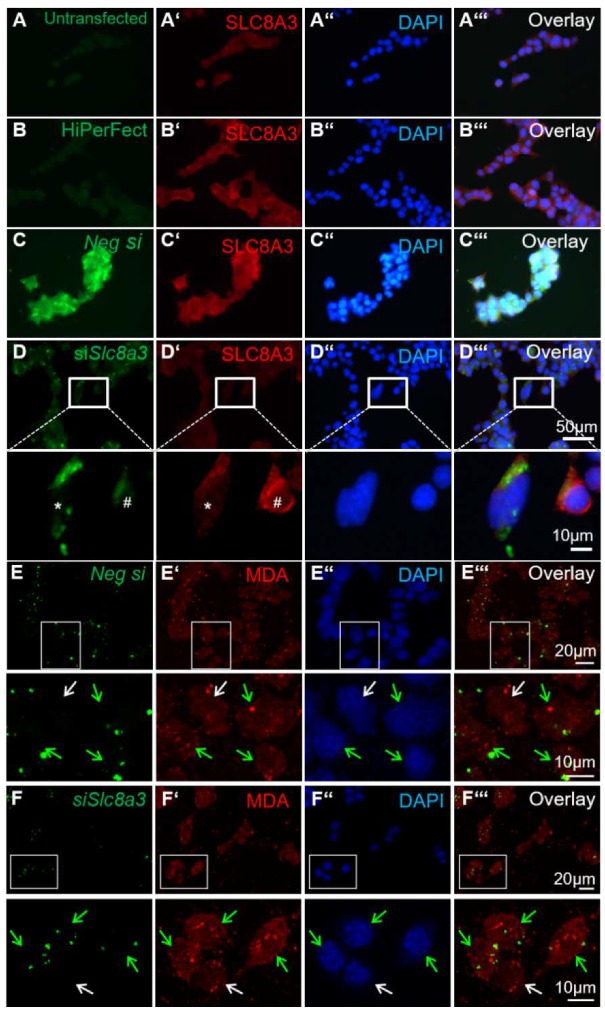
Knockdown of *Slc8a3* increases oxidative stress. (**A**–**D**) Efficiency of SLC8A3 knockdown. SLC8A3 immunofluorescence in MN9D cells that were either non-transfected (A, untransfected), treated with the transfection agent only (B, HiPerFect), transfected with negative *siRNA* (C, green, *Neg si*), or with a specific *siRNA* against *Slc8a3* (green in D). SLC8A3 protein showed no differences between the controls and the cells transfected with negative siRNA but was significantly downregulated in cells transfected with specific *Slc8a3* siRNA (magnification of the white boxed areas). (**E**–**E‴**) Immunofluorescence for the lipid peroxidation marker malondialdehyde (MDA) was comparable between MN9D cells transfected with negative *si*RNA (green arrows) and non-transfected cells (white arrow). (**F**–**F‴**) MDA immunofluorescence was increased in cells transfected with specific *siRNA* against *Slc8a3*. Green arrows indicate successfully transfected cells with specific *siRNA* against Slc8a3, white arrow points to a cell from the same culture that failed to incorporate *Slc8a3 siRNA.*

The existence of crosstalk between Ca^2+^ homeostasis and the redox status in the cells has been well established. To investigate a putative link between loss of function of *Slc8a3* and oxidative stress, we used malondialdehyde (MDA) expression, a known antigen that can be used for the evaluation of cellular reactive oxygen species ROS levels [28], in MN9D cells transiently transfected with *siRNA* against *Slc8a3*. To ensure the specificity of siRNA and efficient knockdown of SLC8A3 protein, first, immunofluorescence for SLC8A3 was performed in untransfected MN9D cells (Figure 6A–A‴), in cells treated only with the transfection reagent HiPerFect (Figure 6B–B‴), and in cells transfected either with Alexa 488-labeled negative *siRNA* (Figure 6C–C‴) or with labeled *Slc8a3*-specific *siRNA* (Figure 6D–D‴). The SLC8A3 labeling intensity was comparable between untransfected (Figure 6A’), and negative-*siRNA*-transfected cells (Figure 6C’). In contrast, SLC8A3 immunofluorescence was nearly abolished in MN9D cells transfected with *siRNA* specific against *Slc8a3* (* in Figure 6D’, magnification of the white boxed area), compared to non-transfected cells (#, in Figure 6D’, magnification of the white boxed area). Moreover, as shown in Figure 6E–6E‴ and the corresponding magnification of the white boxed areas, MDA labeling was comparable in cells transfected with negative *siRNA* (green arrows) and non-transfected cells (white arrows). In contrast, as illustrated in Figure 6F–F‴ and the corresponding magnification of the white boxed areas, in cells transfected with specific *siRNA* against *Slc8a3* (green arrows), MDA immunoreactivity was increased, compared to non-transfected cells (white arrow). These results implicate that knockdown of SLC8A3 increases lipid peroxidation.

### 2.7. Cell-Type-Specific Deletion of TGF-β Signaling is Associated with Oxidative Stress

**Figure 7:**
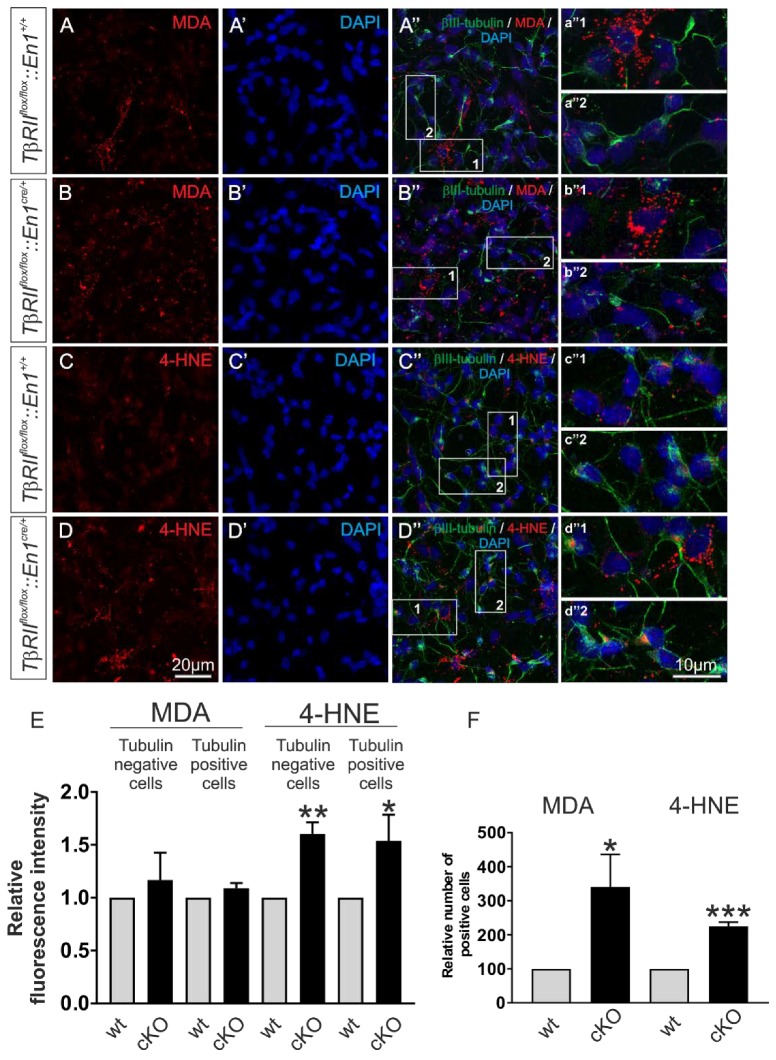
Conditional deletion of TGF-β signaling from Engrailed 1-derived β-III tubulin positive and β-III tubulin negative cells impairs mitochondrial function. (**A**–**D**) Immunofluorescence and subsequent confocal microscopy of the lipid peroxidation markers malondialdehyde (MDA) (**A**,**B**) and 4-hydroxynonenal (4-HNE) (**C**,**D**) in primary ventral midbrain/hindbrain neuronal cultures from *wt* and *TβRII^flox/flox^::En1^cre/+^* (*cKO*) embryos at E14. a″1-d″2 represent higher magnification of the indicated white boxed areas in the respective A″–D″. (**E**,**F**) Quantification of the MDA and 4-HNE fluorescence intensity (E) and of the number of MDA and 4-HNE-positive cells (F) in the mutant (*cKO*), compared to *wt.* Abundance of MDA and 4-HNE was significantly increased in the mutant, compared to *wt* (* *p* < 0.05, ** *p* < 0.01, and *** *p* < 0.001, using the two-tailed unpaired Student’s *t*-test. A total of 345 cells were analyzed from *n* = 3 animals/genotype, the values of *wt* littermates were set to 1.).

To verify whether the results obtained in transfected MN9D cells also apply in En1-derived and TβRII-depleted neurons, expression of the lipid peroxidation markers MDA and 4-hydroxynonenal (4-HNE) [29] was determined in primary neuronal cultures from *wt* and *TβRII^flox/flox^::En1^cre/+^* embryos at E14. Double immunofluorescence for the neuronal marker β-III tubulin and MDA or 4-NHE revealed that in *wt*, both MDA (Figure 7A–A″) and 4-HNE (Figure 7C–C‴) were detected in cultures from r1-derived neurons. As shown in Figure 7A–B″, in primary neuronal cultures from *TβRII^flox/flox^::En1^cre/+^*, the fluorescence intensity for MDA (Figure 7B–B″ and magnification of the white boxed areas b″1 and b″2) was comparable to the *wt* littermate (Figure 7A–A″,E; 1.17 ± 0.25 fold and 1.09 ± 0.05 fold, for β-III tubulin-negative (Figure 7b″1) and β-III tubulin-positive cells (Figure 7b″2), respectively; not significant, using the two-tailed unpaired Student’s *t*-test, total 345 cells (150 cells for MDA and 195 cells for 4-HNE) were examined from *n* = 3 *w*t and *n* = 3 *cKO*). In contrast, the immunofluorescence intensity for 4-HNE was significantly increased in *TβRII^flox/flox^::En1^cre/+^*, for both β-III tubulin-positive (Figure 7D–B″ and high magnification of the white boxed area d″2; Figure 7E; 1.54 ± 0.24 fold; * *p* < 0.05) and β-III tubulin-negative (Figure 7d″1 and 7E; 1.60 ± 0.10 fold; ** *p* < 0.01) cells, compared to *w*t (Figure 7C–C″, c″1 and 2). In addition, the relative number of MDA and 4-HNE-positive cells was significantly increased in cultures derived from *TβRII^flox/flox^::En1^cre/+^*, (Figure 7F; 3.40 ± 0.95 fold and 2.24 ± 1.25 fold, for MDA and 4-HNE, respectively, * *p* < 0.05 and *** *p* < 0.001, using the two-tailed unpaired Student’s *t*-test *n* = 3/per genotype), compared to those from *wt*.

These results strongly suggest that the deletion of TGF-β signaling in En1-derived neurons leads to oxidative stress and mitochondrial dysfunction.

## 3. Discussion

Ca^2+^ homeostasis is a cellular process required for proper cell function and survival, and maintained by the coordinated action of several transporters, among them members of the Na^+^/Ca^2+^ exchanger family. Our results demonstrate that TGF-β plays a role in the regulation of SLC8A3 (NCX3), the isoform 3 of the Na^+^/Ca^2+^ exchanger. Several lines of experimental evidence support this view: In vivo, in mice lacking TβRII in En1-derived cells, SLC8A3 was significantly downregulated (Figure 1), and the expression of SLC8A3 was positively correlated with the expression of the anti-apoptotic protein Bcl-xL (Figure 2) and negatively correlated with the expression of the lipid peroxidation markers MDA and 4-HNE (Figure 6 and Figure 7). In vitro, TGF-β treatment significantly increased SLC8A3 protein expression in neurons (Figure 3) and Smad4 binding to the promoter of *Slc8a3* (Figure 5). 

What could be the biological significance of these results? During CNS development, SLC8A3 is involved in the maturation of oligodendrocytes [15] and in neuronal differentiation [21]. The physiological role of SLC8A3 has also been highlighted in pathophysiological conditions. SLC8A3 expressed in neurons contributes to the maintenance of intracellular Ca^2+^ homeostasis during experimental conditions of excitotoxicity and hyperexcitability and is a well-established player for excitotoxicity-derived Ca^2+^ dysregulation, acting in a neuroprotective manner [30]. In addition, the regulation of Ca^2+^ homeostasis through the upregulation of SLC8A3 activity apparently protects premyelinating oligodendrocytes from ischemic injury [31]. Our results on primary culture from En1-derived TβRII-depleted neurons at E14 implicate that SLC8A3 likely promotes the expression of Bcl-xL (Figure 2A–D), since Bcl-xL was never expressed in *cKO* cells expressing low SLC8A3. These results point to an anti-apoptotic effect of TGF-β signaling in these neuronal populations putatively mediated by the regulation of SLC8A3. Interestingly, certain cells exhibited high SLC8A3 levels, while in others, SLC8A3 expression was low. Based on this observation and taking into consideration that Nestin-positive MN9D cells express lower SLC8A3 levels than β-III-tubulin-positive MN9D cells (Figure 3), it is reasonable to assume that SLC8A3 may be developmentally regulated. During development, TGF-βs may activate distinct signaling pathways, thereby acting pro-apoptotic or anti-apoptotic in a cell-specific and context-dependent manner [32]. Our results add an additional paradigm in this mode of TGF-β action. Whereas TGF-β had no impact on SLC8A3 protein in Nestin-positive cells in vitro, it caused upregulation of SLC8A3 protein in cells expressing the neuronal marker β-III-tubulin, suggesting a differential effect of TGF-β on cells at distinct developmental states.

Whereas these data are straightforward, the results obtained following the inhibition of known signaling pathways used by TGF-β to mediate its effects are more complex (Figure 4). Following inhibition of either the Smad-dependent canonical pathway or the p38 pathway, SLC8A3 in controls was significantly upregulated. In MN9D cells, TGF-β is endogenously expressed, which can act in an autocrine mode through other pathways that may drive a compensatory mechanism with an overshooting effect to counteract the loss of Smad and p38 signaling to maintain basal SLC8A3 levels. In vivo, deletion of TGF-β signaling leads to the downregulation of SLC8A3, since in the mutant mice, all possible TGF-β receptor-mediated signaling pathways are blocked and endogenous TGF-β cannot signal at all. Interestingly, in vivo, a loss of TGF-β signaling cannot be compensated by other endogenously expressed growth factors.

These results are partly in accordance with those obtained in cortical neurons [10], where TGF-β regulates L-type Ca^2+^ channels via JNK1/2, MEK, and p38 signaling. Moreover, p38 has been shown to upregulate basal SLC8A3 levels and, additionally, upon NGF stimulation of PC12 cells [22]. In the present study, inhibition of the JNK or MAPK signaling pathways had no effect on either basal SLC8A3 or on TGF-β-dependent SLC8A3 upregulation.

Whether SLC8A3 is expressed only in the cell membrane or in both the plasmalemmal and mitochondrial membrane has been a matter of controversy. However, increasing experimental evidence supports the view that indeed SLC8A3 is expressed in mitochondria as well. More recently, the expression of both the plasmalemmal and mitochondrial Na^+^/Ca^2+^ exchanger was detected in glia cells [33]. In neurons, the Na^+^/Ca^2+^ exchanger is the major player mediating Ca^2+^ transport back to the cytosol necessary for several neuronal processes, such as neurotransmitter release and synaptic plasticity. Human mesencephalic dopaminergic neurons express both *Slc8a2* and *Slc8a3* in the mitochondria. Specifically, mitochondrial SLC8A3 contributes to mitochondrial Na^+^/Ca^2+^ exchange, and interacts with PINK to regulate Ca^2+^ efflux, thus preventing the neuronal degeneration induced by mitochondrial Ca^2+^ accumulation [24]. Indeed, the role of calcium in the etiology of Parkinson’s disease has been extensively studied and, as an example, PINK1-associated Parkinson’s disease is caused by neuronal vulnerability to calcium-induced cell death [34,35]. Consequently, the blocking of mitochondrial calcium overload has been proposed for therapeutic targeting of oxidative stress and mitochondrial dysfunction in neurodegenerative disorders. In this context, SLC8A2 and SLC8A3 have even been proposed as new molecular targets in Parkinson’s disease and potential targets for therapeutic strategies [24].

Mitochondria are an important site for the formation of secondary lipid peroxidation products, such as MDA and 4-HNE. In the present study, we showed that the knockdown of *Slc8a3* or deletion of TGF-β signaling upregulates the expression of the oxidative stress markers MDA and 4-HNE (Figure 6 and Figure 7). MDA and 4-HNE are the most commonly generated toxic aldehydes, considered to originate under stress conditions, and can react with proteins and/or DNA to form adducts. Depending on their cellular level and the signaling pathways that are able to activate them, MDA and 4-HNE may induce opposite cell responses, such as promote survival or promote cell death. MDA and 4-HNE have been extensively studied and found to be implicated in many pathological processes, such as Alzheimer’s disease, cancer, cardiovascular, and liver disease, as reviewed by several studies [29,36,37]. Notably, excess toxic aldehydes apparently influence the function of tyrosine hydroxylase (TH), leading to impaired dopamine synthesis and dopamine insufficiency [38]. Along this line, MDA and 4-HNE have also been linked to Parkinson’s disease (PD); in substantia nigra neurons from patients with PD, HNE-protein adducts were significantly increased, as detected by immunohistochemistry [39].

Since *Slc8a3*-deficient mice are viable [16], it can be excluded that the decreased *Slc8a3* expression in *TβRII^flox/flox^::En1^cre/+^* mutants by itself leads to the observed [4] neuronal cell death. Nevertheless, the observation of binding of Smad4 to the *Slc8a3* promoter following TGF-β administration in vitro and the fact that cells lacking TGF-β signaling with low SLC8A3 never express Bcl-xL could be a starting point for future studies for a better understanding of the role of TGF-β signaling during development and degeneration of mDA neurons and in 5-HT-associated clinical disorders. To elucidate whether such increased binding of Smad4 to the *Slc8a3* promoter is associated with transcriptional regulation of *Slc8a3,* i.e., whether *Slc8a3* is a direct target of TGF-β, functional studies on *Slc8a3* promoter activity following TGF-β treatment will be necessary. In *TβRII^flox/flox^::En1^cre/+^* cells, the number of MDA and 4-HNE-positive cells was impressively increased, accompanied by an additional significant increase of cellular 4-HNE levels, compared to *wt*. Although we cannot exclude that a loss of TGF-β signaling might have affected other ROS-associated pathways as well, the in vitro data in transfected MN9D cells with specific *siRNA* against *Slc8a3* revealed an upregulation of MDA, implicating that at least in part the data presented in Figure 7 indeed derive from TGF-β-dependent downregulation of SLC8A3 in differentiating dopaminergic and serotonergic neurons.

In summary, within its limitations, the present study provides the first evidence for a role of TGF-β in SLC8A3 regulation in differentiating neurons. We propose a model in which TGF-β signaling somehow (directly or indirectly) maintains and/or affects SLC8A3 expression, which, in turn, together with other signaling pathways, suppresses oxidative stress and promotes the survival of differentiating neurons. Whether *Slc8a3* is a direct target of TGF-β, and the interaction mode between TGF-β signaling and other signaling pathways need to be further elucidated.

## 4. Materials and Methods

### 4.1. Animals

All protocols were carried out in accordance with German ethical guidelines for laboratory animals and approved by the Institutional Animal Care and Use Committee of the University of Freiburg (authorizations: G11/56 (01 July 2011) and X-16/10F,(11 August 2016)). The *T*β*R-II^flox/flox^* mice were generated by Chityl et al. [40] and provided by Dr. Harold Moses (Vanderbilt-Ingram Cancer Center, Nashville, TN, USA). En1-cre mice were provided by Dr. Wolfgang Wurst [41]. Mice with two *T*β*R-II^flox/flox^* alleles and one En1^Cre^ allele were crossbred to yield *T*β*RII^flox/flox^::En1^+/cre^* and have been previously described [4]. 

### 4.2. Genotyping

Genotyping was performed as previously described [4]. For detection of the TβRII wild type (*wt)* or *floxed* gene, the following primers were used: 5′-TAA ACA AGG TCC GGA GCC CA-3′ as the forward primer and 5′-ACT TCT GCA AGA GGT CCC CT-3′ as the reverse primer. The expected band for the floxed gene is 540 bp, while the expected band for the *wt* gene is 420 bp. For detection of the En1-Cre gene, the following primers were used. 5′-GAG ATT TGC TCC ACC AGA GC-3′ as the forward primer, 5′-AGG CAA ATT TTG GTG TAC GG-3′ as the reverse primer for the mutant and 5′-CGA GTC GCG CTG ACT TTT AG-3′ as the reverse primer for the WT. The expected band for the *wt* gene is 250 bp, while the expected band for the Cre gene is 181 bp. PCR was performed with the following cycle conditions: denaturation at 93 °C for 3 min, and 40 cycles of PCR amplification at 93 °C for 30 s and 58 °C for 30 s, and elongation at 72 °C for 30 s, followed by 72 °C for 5 min. PCR products were run on a 2% agarose gel in Tris-acetate-EDTA (TAE) buffer at 100 V, and then photodocumented using a UV transluminator.

### 4.3. Immunohistochemistry

Brains from *wt* and *cKO* embryos were fixed in 4% paraformaldehyde (PFA), cryoprotected, and cut into 10-µm serial coronal sections. Immunohistochemistry was performed as described earlier [42]. Rabbit polyclonal anti-SLC8A3 (1:300) was used as the primary antibody. Goat anti- rabbit-biotin or anti-rabbit IgG coupled either to horseradish peroxidase were used as secondary antibodies.

Diaminobenzidene (DAB) immunohistochemically stained samples were first imaged using the Zeiss Axio Imager M2. Representative images of DAB areas from 5 Engrailed 1-expressing sections were acquired per animal. Visual manual multi-point counting was conducted using ImageJ of each image at the acquired scaled size of 142.10 µm × 106.48 µm (objective 100×; surface area 15,130.808 µm^2^). The counted values of each image at that size were pooled together in the analysis (sum area was 75,654.04 µm^2^). The final step was a unit conversion to mm^2^.

### 4.4. Cell Culture of E14 Ventral Mesencephalon and Ventral Hindbrain

The ventral mesencephalon and ventral hindbrain (corresponding to rhombomere 1) from *wt* and *cKO* mice at E14 was isolated as described earlier [4,42]. The ventral midbrain and ventral hindbrain were dissected from E14 embryos and incubated in 0.25% trypsin for 15 min at 37 °C. Tissue from each littermate was separately dissociated by gentle titration using fire-polished Pasteur pipettes. Dissociated cells were resuspended in high glucose DMEM-F12 medium supplemented with 0.25% bovine serum albumin (BSA), 0.5% N1 additives, 1% glutamin, 0.2% insulin, 33 mM glucose, and 100 U/mL penicillin, 0.5 µg/mL streptomycin, and 100 µg/mL neomycin (PSN; Gibco Life Technologies Thermo Fisher Scientific, 63303 Dreieich, Germany ), and plated onto polyornithin and laminin overnight coated 12-mm^2^ glass cover slips in 24-well plates at a density of 100,000 cells/cover slip. Cells were fixed in 4% PFA and processed for immunocytochemistry.

### 4.5. Cell Culture of MN9D and LUHMES Cells

Lund human mesencephalic (LUHMES), a v-myc-expressing human neuroprogenitor line [26], and MN9D, a hybridoma cell line by fusing embryonic primary cells from the mouse ventral midbrain with cells from the mouse neuroblastoma cell line N18TG2 [27], were used for in vitro experiments. LUHMES cells were cultured on surfaces coated with 0.01% poly-L-ornithine and fibronectin (2 μg/mL, Sigma Aldrich, 82024 Taufkirchen, Germany) in proliferation medium, consisting of DMEM:F12 medium supplemented with 1% N2 supplement and 40 ng/mL FGF2 (R&D Systems; 65205 Wiesbaden, Germany) at a seeding density of 5 × 10^4^ cells/cm^2^. MN9D cells were plated on poly-D-lysine-coated wells or coverslips and cultured in DMEM/F-12, supplemented with 10% FBS, and 1% PSN. Cells were passaged when confluent, and incubated in a 5% CO_2_/95% O_2_ atmosphere at 37 °C. Cells were allowed to differentiate by treatment with 1mM butyric acid for at least 6 days [43]. In undifferentiated and differentiated MN9D cells, serum was retrieved overnight and cells were subsequently treated with recombinant TGF-β (2 ng/mL) (R&D Systems) for 1 h. When the inhibitors of the signaling pathways were used, cells were pre-incubated with the inhibitors for approximately 20 min, before the application of TGF-β. Control and treated cells were either 4% PFA fixed for immunofluorescence, or processed for protein extraction and immunoblotting.

### 4.6. Immunocytochemistry

Immunocytochemistry on primary cultures from the ventral midbrain and hindbrain and on MN9D cells was performed essentially as described earlier [41]. Cells were fixed in 4% PFA/PBS for 30 min at RT, washed with PBS, treated with 1% SDS/PBS for 5 min, blocked with 1% BSA/PBS for 15 min, and incubated with primary antibodies overnight at 4 °C (mouse monoclonal antibodies anti-Nestin 1:100, anti-βIII-tubulin 1:200 (Developmental Studies Hybridoma Bank, Iowa, USA, Cat# Rat-401 and #E7, respectively), rabbit polyclonal anti-SLC8A3 (NCX3; Cat# orb11106, Biozol, 85286 Eching, Germany) 1:100, mouse monoclonal anti-Bcl-xL 1:200 (Cat# sc-136132, Santa Cruz Biotechnology, Dallas, TX, USA), rabbit polyclonal anti MDA 1: 500, and rabbit polyclonal anti-4-HNE 1:500 (Cat# MDA11-S and Cat# HNE11-C AlphaDiagnostics, San Antonio, TX, USA). Cells were washed with PBS, and incubated with either donkey anti-rabbit IgG Alexa Fluor 568 and/or donkey anti-mouse Alexa Fluor 488 at 1:400 1 h at RT. Cells were washed in PBS, mounted with Flouromount-G containing DAPI, and viewed with a Leica TCS SP8 confocal microscope (Leica Microsystems, 35578 Wetzlar Germany). Following double labeling with SLC8A3 and Bcl-xL, cells were scored as those with either high or low SLC8A3 abundance, and the subsequently intensity of Bcl-xL was determined by line scans. Using LAS X software (3.7.0.20979; Leica Microsystems), the threshold was set at maximum intensity of 202; “high SLC8A3” neurons had a maximum intensity of >203 and “low SLC8A3” cells had a maximum value < 201.

### 4.7. Image Acquisition and Analysis

Images were acquired with a Leica TCS SP8 confocal microscope using a CS2 40 × 1.40 oil objective lens. The immunofluorescence intensity for SLC8A3 was determined. Within each experiment, the confocal microscope settings (laser power, detector gain, and amplifier offset) were kept the same for all scans in which the protein expression was compared. Z-stacks of 16–35 optical sections with a step size of 0.5 µm were taken for at least 4 separate fields of view for each experimental condition. Maximum intensity projections were created from the z-stacks. To quantify the protein expression, LAS x software was used to measure the average intensity within the soma. Background substraction was applied to the images. After quantification data were normalized to the mean of controls. Representative images for each figure were processed identically.

### 4.8. Immunoblotting

Protein isolation from MN9D cells, electrophoresis, and blotting procedures were performed as described [9]. Protein concentration was determined by a Thermo Scientific NanoDrop 2000 spectrophotometer (absorbance at 280 nm). Primary antibodies were diluted: Rabbit polyclonal antibody anti- SLC8A3 (NCX3 Cat# orb11106, Biozol,) 1:1000, rabbit polyclonal anti-TβRII (C-16) 1:1000 (Cat# Sc-220, Santa Cruz Biotechnology, Dallas, TX, USA), and mouse monoclonal anti-GAPDH 1:20,000 (Cat# ab8245, Abcam, Cambridge, UK). Blots were developed in enhanced chemiluminescence reagents, and signals were visualized on X-ray films. Films were scanned and the signal ratio protein of interest:housekeeping gene was quantified densitometrically. Differences in the signal ratio were tested for significance using two-tailed unpaired Student’s *t*-test. Results with levels of * *p* < 0.05 were considered significant.

### 4.9. Chromatin Immunoprecipitation Assay

Chromatin immunoprecipation (ChIP) assays and PCR were performed on control and TGF-β-treated LUHMES cells using an EZ ChIP kit (Merck Millipore, Darmstadt, Germany), as described [9] and following the manufacturer’s instructions. Briefly, DNA was crosslinked to protein with 1% paraformaldehyde. Cell lysates were obtained by scraping followed by sonication (Diagenode, Bioruptor Sonication Device; Ougreé, Belgium) to shear cellular DNA. Overnight immunoprecipitations were performed with histone 3 (mouse monoclonal anti-H3; Cat # MABE923, Merck, 64293 Darmstadt, Germany), IgG (mouse polyclonal Cat# 12-371 from Merck-Millipore), and anti-Smad4 (rabbit polyclonal, Cat# 38454, Cell Signaling, 60314 Frankfurt, Germany) antibody. On the next day the crosslinks were reversed, and bound DNA was purified. Subsequently, PCR was performed using primers specific for Smad4 promoter sequences in human SLC8A3. Primer sequences (with their starting positions in the promoter sequence; NG047080) were: Smad4 forward: 5′-CGAGGAGCGTTCTGAGAGTC-3′, Smad4 reverse: 5′-GCGGAGAGGCTGGTTTCTG-3′. The SLC8A3 promoter sequence was retrieved using EnsEMBL, and the conserved Smad4 binding sequence was identified by sequence alignment using ClustalW (EMBL-EBI).

### 4.10. Transient Transfection of MN9D Cells

MN9D cells were plated on 12-mm^2^ glass cover slips in a density of 50,000 cells per well 24 h prior to transfection. Cells were transiently transfected with Alexa 488-labeled siRNA specifically targeting mouse *Slc8a3* mRNA (purchased from Qiagen, 40724 Hilden, Germany) using HiPerFect reagent (Qiagen), following the manufacturer’s instructions as previously described [44]. Briefly, 30 µL of HiPerFect and 1 mL of medium without FBS and PSN were thoroughly vortexed before 10 nM siRNA was added. To allow the formation of HiPerFect-siRNA complexes, the mixture was incubated in the dark for 30 min at RT before being cautiously pipetted onto the cells. A sequence that revealed no homology with any known mammalian gene was labeled with Alexa 488 (AllStars Negative Control siRNA, Qiagen) and was used as a control, i.e., negative siRNA. Then, 24 h following transfection, cells were processed for immunofluorescence.

### 4.11. Statistics

Data are presented as the mean ± standard error of mean (SEM). Statistical analysis was performed using the two-tailed unpaired Student’s *t*-test or one-way ANOVA and Bonferroni post-hoc test. Differences were considered statistically significant at * *p* < 0.05, ** *p* < 0.01, and *** *p* < 0.001.

## Supplementary Materials

Supplementary materials can be found at https://www.mdpi.com/1422-0067/21/8/2735/s1.

## Abbreviations

4-HNE	4-hydroxynonenal
5-HT	5-hydroxytryptamine
Bcl-xL	B-cell lymphoma-extra large
BDNF	Brain derived neurotrophic factor
cKO	Conditional knockout
DMEM	Dulbecco’s Modified Eagle Medium
DR	Dorsal raphe
E#	Embryonic day
En1	Engrailed 1
ERK1/2	Extracellular signal–regulated kinases-1/2
GAPDH	Glyceraldehyde 3-phosphate dehydrogenase
H3	Histone 3
JNK1/2	c-Jun N-terminal kinases
L-type Ca^2+^	Long-lasting calcium channel
LUHMES	Lund Human Mesencephalic
MAPK	Mitogen-activated protein kinase
MEK	Mitogen-activated protein kinase kinase
mDA	Midbrain dopaminergic
MDA	Malondialdehyde
NCX3	Na^+^/Ca^2+^ exchanger 3
NGF	Nerve growth factor
PC12	Pheochromocytoma of the rat adrenal medulla
PD	Parkinson’s disease
PINK1	PTEN-induced kinase 1 or PTEN-induced putative kinase 1
ROS	Reactive oxygen species
siRNA	Silencing RNA
SLC8A3	Solute carrier Family 8 member A3
Smad	Small mother against decapentaplegic
SNc	Substantia nigra pars compacta
TGF-β	Transforming growth factor beta
TRPC	Transient receptor potential canonical channels
TβRII	Type II TGF-β receptor
vH	Ventral hindbrain
VTA	Ventral tegmental area
Wt	Wildtype
