# Supplementary figures and images for: TGF-β Signaling Regulates SLC8A3 Expression and Prevents Oxidative Stress in Developing Midbrain Dopaminergic and Dorsal Raphe Serotonergic Neurons
Source: Int J Mol Sci. 2020 Apr 15;21(8):2735. doi: 10.3390/ijms21082735 (PMC7216069; doi:10.3390/ijms21082735)

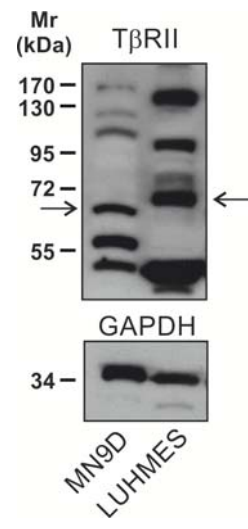

Supplement: Supplementary file 1 [file ijms-21-02735-s001.pdf]
